# Supplementary material for: Acceptance and compliance with micronutrient powder (MNP) among children aged 6–23 months in northern Nigeria
Source: PLOS Glob Public Health. 2022 Oct 17;2(10):e0000961. doi: 10.1371/journal.pgph.0000961 (PMC10022258; doi:10.1371/journal.pgph.0000961)
Supplement: S1 Dataset — (ZIP) [file pgph.0000961.s008.zip › S8_Dataset/Direct Observations.zip!/Direct Observations/070615_K_J_AD_2_DO.pdf]

1.1.1 Instrument F –Direct Observation Form

<<Instrument F Direct Observation Form>>

PI Name: PragyaMathema,Stephen Kodish
Study Title: Formative research to develop an infant and young child feeding (IYCF) intervention with micronutrient powder (MNP) for children aged 6 – 23 months
PI Version/Date: Version1.0/January 24, 2015

Date: 07/07/15Location: Hillinde

Data Collector: Amina Duniya

Main goals of this direct observation are to answer the following questions:

1. In this household, what would be the best way(s) to give nutritional supplements to young children? Why?

2. In this household, describe the food sharing that you observe throughout the meal. Are there any predictable patterns?

3. In this household, who is favored and how is this favoritism expressed during meal preparation and eating?

Household Composition Table (Fill out as you do data collection)

INITIALS	SEX (M/F)	AGE (estimated)	Relationship to household	Description (not necessary)
BM	F	25years	Mother	Slim and dark in complexion, wearing a bead on her neck and tying a multi colored wrapper
GM	F	50years	Grandmother	Smallish and dark in complexion, tying a blue lace wrapper and beads on her neck and right hand
SS	F	3years	Senior sister of BB	Dark in complexion, wearing a green gown
BB	M	19months	Baby (index child)	Dark and chubby, no cloth on his body, wearing a bead on his neck

PARTICIPANT ID: _________

TIME	ACTOR INITIALS	RECIPIENT INITIALS	WHAT HAPPENED	FOOD	Reactivity
8:30	BM		Pounding guinea in a mortar		
	BB		Sitting on the floor close to BM and playing		
8:40	BB		Still playing		
	BM		Still pounding		
8:50	BB		Starts crying		
	BM	BB	Picks him up and straps him to her back		
	BM		Continues pounding		
9:00	BM		Still pounding with BB strapped to her back		
9:05	BB		Begins to cry		
	BM	BB	Brings him down and gives him food to eat	Tuwo and kuka	
9:10	BM		Fetches water and washes the guinea corn		
9:20	BM		Finished washing the guinea corn and begins to wash plate		
	BB		Still eating	Tuwo and kuka	
9:30	SS		Comes in and tries to take BB's tuwo	Tuwo and kuka	
	BM	SS	Stops her from eating BB's food and gives her kunu to drink		
9:40	BB		Still eating	Tuwo and kuka	
9:50	BB		Stops eating and starts playing with food		

TIME	ACTOR INITIALS	RECIPIENT INITIALS	WHAT HAPPENED	FOOD	Reactivity
9:55	BB		Still playing with food		
	BM		Washing dishes and looking at BB		
10:05	BB	BM	Gets tired of playing with food and gives BM the plate		
10:15	BM	BB	Gives him water to drink		
	BM	BB	Washes his body with water		
10:25	BB		Begins to cry		
	BM	BB	Brest feeds baby	Brest milk	
	BB		Finished breast feeding		
10:30	BB		Begins to drink kunu gero	Kunun gero	
10:40	BB		Still drinking kunun gero	Kunun gero	
10:45	BB		Finished drinking kunu	Kunun gero	
	BB		Begins to play with BM		
10:50	SS	BB	Starts playing with BB		
11:00	SS	BB	Still playing together		
	BM		Talking with GM		
11:05	SS	BB	Still playing together		
11:10	SS		Starts sleeping		
11:15	BB		Continues to play by himself		
11:20			Still playing		
11:25	BM		Opens up the MNP and pours into a little quantity of cold kunu	Kunu and MNP	

TIME	ACTOR INITIALS	RECIPIENT INITIALS	WHAT HAPPENED	FOOD	Reactivity
	BM	BB	Gives it to BB to drink	Kunu mixed with MNP	
	SS	BB	Wakes up and sees baby drinking kunu and begs for some	Kunu mixed with MNP	
	BB		Refuses to give to SS and continues drinking kunu		
	BM	SS	Tells SS it is not meant for her		
11:30	BB		Finished drinking the kunu		
	SS		Picks up the plate and licks the remaining kunu from the plate		
	BB		Continues to play		
11:40	BB		Still playing		
11:45	BM	BB	Breast feeds BB	Breast milk	
	BB		Starts sleeping		
11:50	BB		Still sleeping		
12:00	BB		Still sleeping		
12:05	BB		Still sleeping		
12:10	BB		Still sleeping		
	BM		Talking with GM		
12:20	BB		Still sleeping		
12:30	BM		Joins baby and starts to sleep		
12:40	BB		Still sleeping with BM		
12:50	BB		Still sleeping with BM		

TIME	ACTOR INITIALS	RECIPIENT INITIALS	WHAT HAPPENED	FOOD	Reactivity
1:00	BB		Still sleeping with BM		
1:10	BB		Still sleeping with BM		
1:20	BB		Still sleeping with BM		
1:25	BB		Still sleeping with BM		
1:30	BB		Still sleeping with BM		
1:35	BM		Wakes up		
1:40	BB		Still sleeping		
1:45	BB		Still sleeping		
	BM		Pounding guinea corn		
1:55	BB		Still sleeping		
	BB		Wakes up		
2:00	BM	BB	Breast feeds BB	Breast milk	
2:05	BB		Finished sucking	Breast milk	
	BM	BB	Plays with BB		
2:10	BM	BB	Still playing together		
2:20	BM	BB	Gives him fura da nono to drink	Fura da nono	Giving him too much food and it doesn’t seem natural
2:25	BB		Still drinking fura da nono	Fura da nono	
2:30	BB		Still drinking fura da nono	Fura da nono	
	BB		Finishes and starts to play		

TIME	ACTOR INITIALS	RECIPIENT INITIALS	WHAT HAPPENED	FOOD	Reactivity
2:40	BB		Still playing		
2:45	BB		Drinking fura da nono	Fura da nono	
2:50	BM	BB	Washes him with water		This action doesn't seen natural
2:55	BM	BB	Breast feed BB	Breast milk	
	BM	BB	Finished breast feeding and starts playing with BB		
3:00	SS	BB	SS plays with BB		
3:05	BB		Still playing with SS		
3:10	BB		Starts crying and BM picks him up		
	BM	BB	Straps BB to her back		
3:15	BM		Pounding guinea corn		
	BB		Strapped to BM's back		
3:20	BM	BB	Puts him on the floor to play		
3:30	BB		Still playing		
3:35	BM	BB	Gives him boiled moringa leaves to eat	Moringa leaves	
3:40	SS	BB	Plays with BB		
3:45	SS	BB	Still playing together		
3:50	BB		Playing alone		
3:55	BM	BB	Breast feeds him	Breast milk	
	BB		Finished sucking and starts playing		
4:00	BB		Still playing		

TIME	ACTOR INITIALS	RECIPIENT INITIALS	WHAT HAPPENED	FOOD	Reactivity
4:05	BB		Still playing		
4:10	BB	BM	Crawls to BM to breast feed him	Breast milk	
4:15	BB		Still breast feeding	Breast milk	
4:20	BB		Starts playing		
	BM		Watching BB Play		

Primary Questions (Complete after you complete data collection):

From what you have seen observing THIS HOUSEHOLD, please answer the following questions in detail:

Question	Write your detailed response here
1. In this household, what would be the best way(s) to give nutritional supplements such as an MNP to young children to ensure compliance?<br><br>Why?	It is the mother because she is actively involved in the feeding of the child
2. In this household, who is favored during meal preparation or feeding?	The baby is highly favored because the mother guides him during meal time

How is this favoritism expressed?	
3. How could we promote the MNP supplement for this family that would attract their attention?<br><br>Who would they listen to?	By enlightening them about the benefits and what the child stands to gain when he is fed with the MNP<br><br>The most effective person they will listen to is the village head
4. In this household, what would be the best ways to promote the MNP but limit how much it would be shared?<br><br>Why?	By telling the parent what the child stands to lose if another child that the product is not meant for takes it
5. What other issues and concerns did you see with this family that are important for us to consider with regard to introduction of an MNP?<br><br>Why?	Because the mother always monitors his feeding

6. Based on your experience observing this child today, do you believe reactivity negatively impacted your ability to see the food-related behaviors of the child?<br><br>Please explain your answer in detail.	No I don’t think so because the mother was just trying to show that she feeds her child well
